# Mesenchymal stem cell conditioned medium alleviates oxidative stress injury induced by hydrogen peroxide via regulating miR143 and its target protein in hepatocytes

**DOI:** 10.1186/s12865-017-0232-x

**Published:** 2017-12-19

**Authors:** Xuejing Xu, Dong Li, Xue Li, Qing Shi, Xiuli Ju

**Affiliations:** 1 Shenzhen Research Institute of Shandong University, Shenzhen, 518057 China; 2 Department of Pediatrics, Qilu Hospital, Shandong University, Jinan, 250012 China

**Keywords:** Hepatocyte, Oxidative stress injury, Mesenchymal stem cell, miRNA, Apoptosis

## Abstract

**Background:**

To investigate the impact of miRNA (microRNA) on hepatic oxidative stress damage under the human mesenchymal stem cell conditioned medium (MSC-CM) and explore the roles of the beta-1 adrenergic receptor (ADRB1) and hexokinase 2 (HK2) in this process.

**Methods:**

Hydrogen peroxide was used to induce oxidative stress injury in the human normal liver cell line L02. MSC-CM was separately prepared. After treatment with MSC-CM, the protective effects of MSC-CM on oxidative stress injury were assessed by changes in apoptosis, cell viability, cell cycle, and mitochondrial membrane potential. According to the microarray analysis, 19 disparately expressed miRNAs were selected for RT-PCR and miR143 identified as having significant differential expression in MSC-CM against oxidative stress injury. Subsequently, the predicted target proteins of miR143 were selected by bioinformatics software, and verified by western blot. In addition, down-regulation and up-regulation of miR143 expression and hydrogen peroxide induced hypoxia injury were carried out on L02 cells to study the role of miR143.

**Results:**

MSC-CM significantly attenuated H_2_O_2_ induced oxidative stress injury. The expression of miR143 was increased following oxidative stress injury whereas it decreased after MSC-CM treatment. The expression levels of HK2 and ADRB1 regulated by miR143 and Bcl-2 decreased under H_2_O_2_ treatment but were restored following MSC-CM treatment. However the expression levels of Bax and BMF increased after H_2_O_2_ injury and decreased after MSC-CM treatment. Moreover over-expression or down-regulation of miR143 aggravated or alleviated hepatocyte apoptosis respectively.

**Conclusions:**

MSC-CM may alleviate H_2_O_2_ induced oxidative stress injury by inhibiting apoptosis and adjusting miRNA expression. Moreover down-regulation of miR143 protects L02 cells from apoptosis and initiates an adaptive process by adjusting the expression of HK2 ADRB1 and apoptosis-related proteins.

**Electronic supplementary material:**

The online version of this article (10.1186/s12865-017-0232-x) contains supplementary material, which is available to authorized users.

## Background

Oxidative stress injury leads to severe local and remote tissue injury and subsequent distant organ functional failure, including that of the gastrointestinal tract, cardiovascular system, brain, and liver [1–4]. The mechanisms of hypoxia are mainly derived from cellular damage [5, 6]. Until now, no effective therapeutic protocol has been proven to modify the course of oxidative stress injury.

Substantial previous work has demonstrated that mesenchymal stem cells (MSCs) have notable capability to repair ischemic organ tissue [7]. Studies have shown that MSC conditioned medium (MSC-CM) is a promising novel therapeutic agent to promote proliferation and anti-apoptotic and anti-inflammatory responses after acute organ injury [8–13]. The rationale for MSC therapy is increasingly recognized to act by a secretion (paracrine) rather than differentiation mechanism [14–16].

MicroRNAs (miRNAs) play critical roles to regulate differentiation, paracrine activity, and other cellular activities [17]. MiRNAs are small non-coding RNA molecules that affect mRNA stability and translation, which play a key role in mediating biological function due to their prominent role in gene regulation. Mature miRNAs are important for numerous cellular processes and deregulation of miRNAs can be observed in several pathological processes [18]. Until now, despite the extensive and increasing knowledge regarding the regulation of miRNAs, little is known about the expression change of miRNAs in hepatocytes during the MSC-CM mediated apoptosis rescue process. In the present study, we demonstrate that MSC-CM is able to ameliorate hepatocyte injury. Nineteen chip hybridization-screened miRNAs were verified to show significant changes in expression during this process, from which miR143 was verified by quantitative PCR. The predicted target proteins of miR143, HK2 and ADRB1, were analyzed by three bioinformatics algorithms, and verified by western blot. We also down-regulated and up-regulated miR143 expression to study the effect of miR143 on apoptosis, cell cycle, mitochondrial membrane potential (MMP), and cell viability in L02 cells.

## Methods

### Culture of human umbilical-cord-derived MSCs

Human umbilical cord was obtained from healthy donors at the Department of Obstetrics in Shandong University Qilu Hospital, Jinan, China. Umbilical cords were dissected to remove blood vessels and cut into small fragments. Then, human umbilical-cord-derived MSCs (hUC-MSCs) were cultured in α-minimum essential medium (α-MEM; Gibco; Thermo Fisher Scientific, Waltham, MA, USA) with 10% fetal bovine serum (FBS; Gibco) supplemented with 100 U/mL penicillin and 100 μg/mL streptomycin (Gibco). Cultures were maintained at 37 °C in a humidified atmosphere of 5% CO_2_ in air. The culture medium was changed every 3–4 days. The MSCs used throughout this study were between passage 3 and 6.

### Identification and differentiation of hUC-MSCs and preparation of MSC conditioned medium

Flow cytometry analysis was performed to characterize MSCs according to the previously study [19]. The hUC-MSCs were analyzed for their ability to differentiate into adipocytes and osteoblasts as described previously [20]. The cells used were between passage 5 and 7.

Once the MSCs reached 70-80% confluency, the medium was replaced with fresh full medium and harvested after 24 h. Subsequently, MSC-CM were centrifuged at 2500 rpm for 20 min with 0.1-μm filtration to remove detached MSCs and cell debris.

### L02 cell culture and processing

The human hepatic cell line L02 [21] was purchased from the Cell Resource Center of the Shanghai Institutes for Biological Sciences, Chinese Academy of Sciences (Shanghai, China). L02 cells were cultured in RPMI-1640 medium (Gibco) with 20% FBS (Gibco),100 U/mL penicillin, 100 μg/mL streptomycin (Gibco), and 10 μg/mL insulin (Wanbang, Jiangsu, China) at 37 °C,in a 5% CO_2_ incubator [21]. Subsequently, the L02 cells were cultured to 70–80% confluency before exposure to 1 mM hydrogen peroxide (H_2_O_2;_ DAMAO, Tianjin, China.) for 3 h to induce oxidative stress injury. In detail, L02 were randomly divided into the following three groups: the normal control group (Control) with normal incubation, the H_2_O_2_ treatment group (H_2_O_2_) which were incubated with 1 mM H_2_O_2_ for 3 h in the above medium, and the rescue group with addition of 20% MSC-CM for 6, 24, and 48 h following H_2_O_2_ (H_2_O_2_ + MSC-CM). Cell Counting Kit-8 (CCK-8; DOJINDO, Kyushu, Japan) was used to find the MSC-CM optimal time, which was determined to be 24 h. All cell experiments were replicated a minimum of five times.

### Apoptosis, cell cycle, and MMP detection by flow cytometry

To assess apoptosis, flow cytometry with AnnexinV/Propidium iodide (PI) double staining (BD Biosciences, New Jersey, United States) was used. Cell cycle stage (70% ethanol fixed 12–24 h at 4 °C) and MMP were also evaluated by flow cytometry with Propidium iodide (PI) staining (Beyotime, Shanghai, China) and JC-1 staining (1st J-aggregate-forming cationic dye; BD Biosciences), following the manufacturer’s instructions. Apoptosis, cell cycle stage, and MMP were detected using flow cytometry (Millipore, Billerica, MA) and the data were analyzed using Guava Incyte (version 2.8; EMD Millipore).

### Quantitative RT-PCR analysis of miRNAs and miR143 selection

SD rats were purchased from the Experimental Animal Centre of Shandong University and housed in a specific pathogen-free (SPF) animal facility. Hepatic cirrhosis in SD rats was induced with carbon tetrachloride subcutaneous injection and alcohol oral administration. hUC-MSCs or PBS were transplanted by intravenous injection once per week for 4 weeks. In detail, rats were randomly divided into the following three groups: normal rats (Nor) with PBS, cirrhotic rats (CTr), and cirrhotic rats with MSC (MSC). The gene expression alterations were calculated between the PBS and MSC groups by miRNA microarray analysis.

Liver total RNA was harvested using TRIzol (Invitrogen) and the miRNeasy mini kit (QIAGEN) according to the manufacturer’s instructions. After quantification using the NanoDrop 1000, the samples were labeled using the miRCURY™ Hy3™/Hy5™ Power labeling kit and hybridized on the miRCURY™ LNA Array (v.18.0). Following the washing steps, the slides were scanned using the Axon GenePix 4000B microarray scanner.

Scanned images were then imported into GenePix Pro 6.0 software (Axon) for grid alignment and data extraction. miRNA replicates were averaged and the normalization factor calculated for miRNAs with intensities ≥30 in all samples. Expressed data were normalized using the median normalization. After normalization, differentially expressed miRNAs were identified through Fold Change filtering. Finally, hierarchical clustering was performed to show distinguishable miRNA expression profiling among samples.

Nineteen disparately expressed miRNAs (Additional file 1) were selected by gene chip hybridization in the model of cirrhotic rats treated with MSC. miRNA was extracted from the L02 cells, which were treated with MSC-CM for 24 h after 1 mM H_2_O_2_ treatment for 3 h using the miRcute miRNA Isolation Kit (Tiangen, Beijing, China) according to the manufacturer’s instructions. To verify the expression of the specific miRNAs in the process of H_2_O_2_ injury and the protective effect of MSC-CM, RT-PCR was performed on the 19 disparately expressed miRNAs described above.

First-strand cDNA of miRNA was synthesized using 0.25 μl of miRNA in a 20 μl reverse transcriptase reaction mixture using the miRcute miRNA First-Strand cDNA Synthesis Kit (Tiangen) with miRNA specific primers (Tiangen). Using the miRcute miRNA qPCR Detection (SYBR Green) kit (Tiangen), quantitative RT-PCR was performed with the ABI Prism 7500 sequence detection system. The miRNA expression levels were normalized to those of U6. Data were analyzed using Sequence Detection Software (Version 1.4, Applied Biosystems, Carlsbad, CA). The relative quantity of the transcript was calculated using 2^-ΔCt^ the method, where ΔCt = Ct_miRNAtest_-Ct_miRNActr._.

### Bioinformatics of miR143

We used three algorithms, namely miRBase (http://www.mirbase.org/), TargetScan (http://www.targetscan.org/), and PicTar (http://pictar.mdc-berlin.de/) to predict the corresponding downstream target genes of the miRNA143.

### Cell transfection and miR143 treatment

To study the effect of miRNA143 in oxidative stress injury, we up- and down-regulated miRNA143 by transfecting miR143 mimics, miR143 inhibitors, a negative control (NC), or an miRNA inhibitor negative control (inhibitor NC, GenePharma). One day before transfection, L02cells were plated in 24-well plates without antibiotics so that they would be 30–50% confluent at the time of transfection. Cells were transfected with 33 nM miRNA oligomer using Lipofectamine 2000 (Invitrogen) according to the manufacturer’s instructions; the culture medium was replaced at 6 and 36 h after miRNA transfection. After 36 h of transfection, the cells were used for RT-PCR. The RT-PCR analysis demonstrated that miR143 inhibitors significantly down regulate miR143 expression in L02 cells compared with inhibitor NC and NC. On the other hand, miR143 mimics significantly up regulated miR143 expression, and there was no difference between inhibitor NC and NC. According to the analysis of RT-PCR, miR143 was transfected and differently expressed into L02 cell successfully (Fig. 6c).

L02 cells were incubated at 37 °C for 36, 48, and 60 h. CCK8 was used to find miR143 transfected time. CCK-8 was used to detect cell proliferation. Briefly, after transfection with miR143 mimics, inhibitors, or scrambled oligonucleotides (miRNA negative control-NC and miRNA inhibitor NC; 33 nM; Gene Pharma, Shanghai, China) using Lipofectamine 2000 (Invitrogen, Carlsbad, CA), the cells were incubated with 0.8 mM H_2_O_2_ for 2 h, then 10 μl CCK-8 was added. After 2–3 h, the optical density (OD) was measured with a microplate reader (KeyGen, NanJing, China) at 450 nm using the following formula: OD_experiment_ - OD_blank_ (blank control group: no cells and medium). According to the analysis of CCK8, the optimal miR143 transfection time was 60 h. All cell experiments were replicated a minimum of five times.

Subsequently the cells were treated with 0.8 mM H_2_O_2_ for 2 h. Apoptosis, cell cycle, cell viability, MMP, and western blot analysis were performed for recovery following H_2_O_2_ injury.

### Western blotting

L02 cell lysates (1 μg/μl, Beyotime) were prepared using RIPA buffer containing protease inhibitor PMSF (1 mM,Beyotime). Total proteins were separated by 12% SDS-PAGE. The proteins were then transferred onto polyvinylidene difluoride membranes (Millipore, USA). After blocking with 0.5% skimmed milk powder solution for 70 min at room temperature, proteins were detected by western blotting with the indicated antibodies overnight. Antibodies against HK2 (1:1000, Cat: 60,004–1-lg), Bcl-2 (1:1000, Cat: 12,789–1-AP), and Bax (1:2000, Cat: 60,267–1-lg) were purchased from Proteintech (Wuhan, China), antibodies against ADRB1 (1:1000, ab3342) and recombinant human Bcl2 modifying factor (BMF, 1:500, ab181148) were purchased from Abcam (Cambridge, MA USA). After washing with TBST, the membranes were incubated with secondary antibodies (1:10,000, Goat Anti-Mouse IgG (H + L), Cat: SA00001–1; 1:10,000, Goat Anti-Rabbit IgG (H + L), Cat: SA00001–2; proteintech) and developed using ECL (Millipore, Billerica, MA). The quantified densitometric analysis was performed using the UVP BioSpectrum multispectral imaging system (UltraViolet Products Ltd., Upland, CA, USA). The relative levels of the proteins were then determined by measuring the intensity of the corresponding bands. All the western-blot analyses were repeated a minimum of five times and the values were normalized to the constitutive expression of GAPDH.

### Statistical analysis

Quantitative data are presented as the mean ± standard deviation of a minimum of five independent replicates for of H_2_O_2_ injury, MSC-CM treatment, and miR143 transfection. For comparisons between miR143 mimics and inhibitor group under H_2_O_2_ injury, independent two sample *t*-tests were performed. For comparisons among the various cell lines, one-way analysis of variance (ANOVA) with Tukey-Kramer postdoc analysis was employed. Differences between the values were considered to be significant at *P* < 0.05. All the statistical analysis were carried out with SPSS 20.0 software program (SPSS, Inc., Chicago, IL, USA) and graphs were made using GraphPad Prism 6 (Beijing, HuanZhongRuiChi).

## Results

### In vitro differentiation of hUC-MSCs

**Fig. 1:**
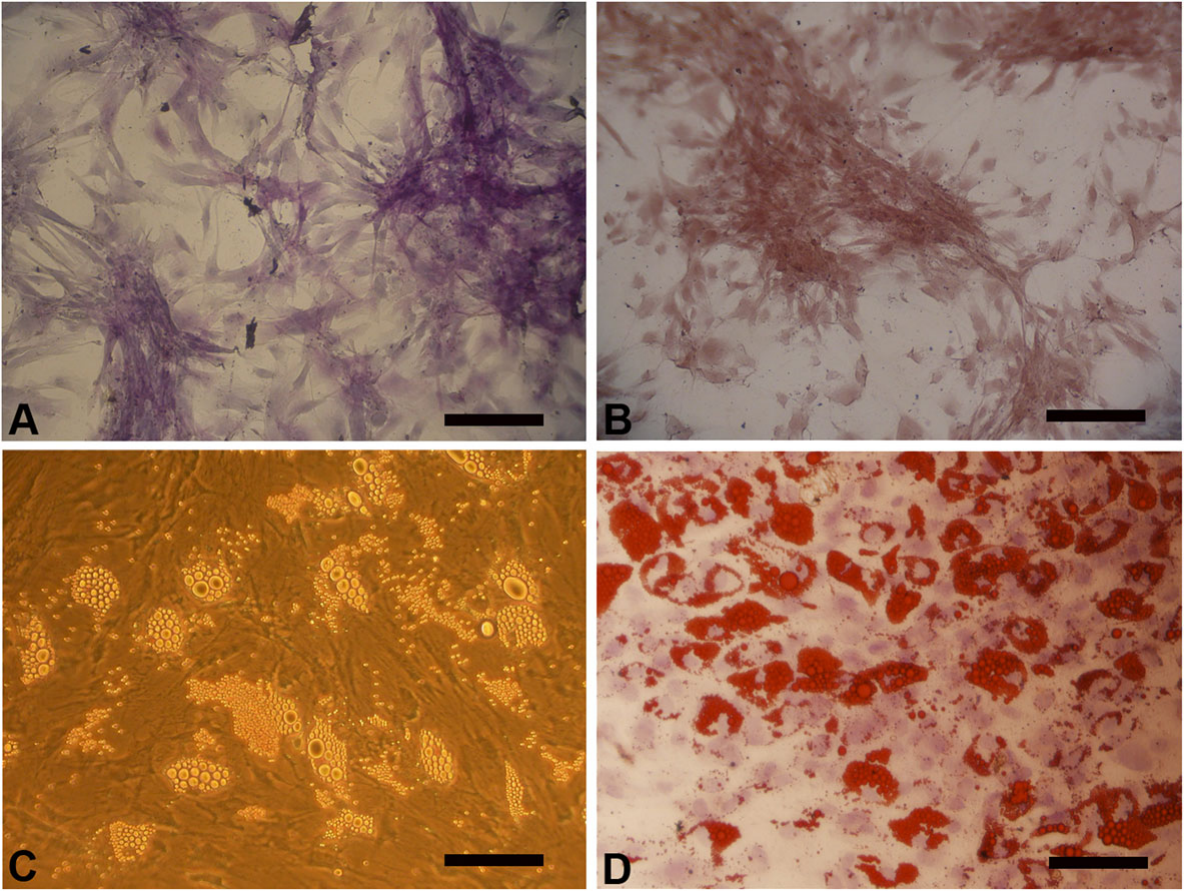
Differentiation of hUC-derived MSCs. Osteogenic differentiation of MSCs was demonstrated by the formation of calcium-hydroxyapatite-positive areas stained in red by Alizarin Red (**a**) and brown by alkaline phosphatase (**b**). Adipogenic differentiation was demonstrated by intracellular lipid vacuoles stained in red by Oil Red O (**c**-**d**). Scale bar = 100 μm

For osteogenic differentiation, the cells were cultured for 20 days in osteogenic medium. Calcium precipitation, determined by alkaline phosphatase and Alizarin Red S staining, was observed in the hUC-MSCs (Fig. 1a–b). Adipogenic differentiation of hUC-MSCs was apparent after 14 days. Oil Red O-positive lipid droplets were observed (Fig. 1c–d).

### Protective effect of MSC-CM on apoptosis of L02 cells exposed to H_2_O_2_

**Fig. 2:**
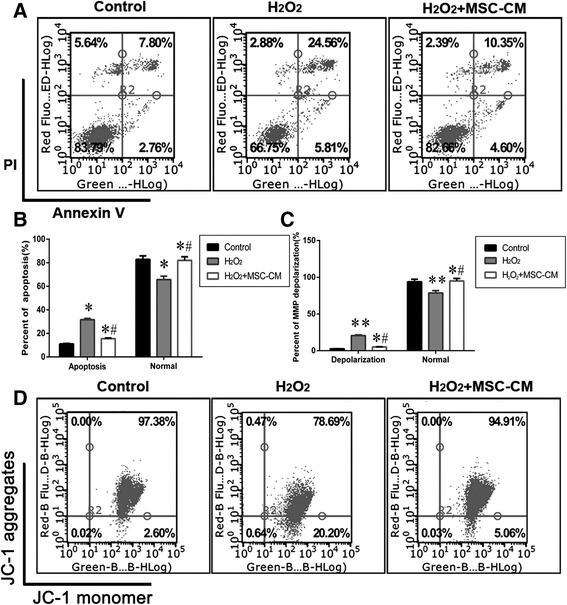
MSC-CM treatment decreases apoptosis. H_2_O_2_: L02 incubated with 1 mM H_2_O_2_ for 3 h; H_2_O_2_ + MSC-CM: rescue group with addition of 20% MSC-CM for 24 h following H_2_O_2_ injury. **a**. MSC-CM protects L02 cells from apoptosis examined by Annexin V/PI double staining and flow cytometry. **b** The percentage of apoptotic L02 cells in the total cell population. **c**-**d**. MSC-CM protects L02 cells from MMP depolarization examined by JC-1 staining and flow cytometry. The lower right quadrant: MMP depolarization, a sign of early apoptosis (Mean ± SD. *n* = 10. * *P* < 0.05 and ** *P* < 0.01 vs. control group; # *P* < 0.05 vs. H_2_O_2_ group)

To quantify the effects of MSC-CM (20% MSC-CM for 24 h) on H_2_O_2_-induced (1 mM H_2_O_2_ for 3 h) cell apoptosis and necrosis, we performed flow cytometry with AnnexinV/PI double staining, which was used to discriminate apoptosis from necrotic cells, JC-1 was employed to detect MMP (∆ψ), PI single staining was applied to detect cell cycle and CCK-8 analysis was applied to demonstrate that the MSC-CM could prevent L02 cell from undergoing apoptosis. According to Annexin V/PI flow cytometry analysis (Fig. 2a–b, *P* < 0.05), the percent of typical apoptotic cells reached 31.6% ± 1.07% and 15.58% ± 0.5 5% in the H_2_O_2_ and H_2_O_2_ + MSC-CM groups respectively, while the apoptosis rate of the control group was 11.04% ± 0.39%. Compared with the H_2_O_2_ group, the apoptosis rate of normal cells increased by 16.31% ± 3.26% (65.69% ± 2.91% vs. 82.00% ± 3.11%, Fig. 2a–b, *P* < 0.05) in the H_2_O_2_ + MSC-CM group. In addition, the percentage of apoptotic cells whose MMP decreased visualized by JC-1 staining clearly increased after H_2_O_2_ stimulation, and was restored after MSC-CM treatment (Fig. 2c–d, *P* < 0.05).

**Fig. 3:**
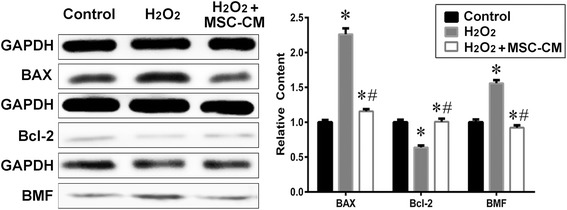
Apoptosis-related protein relative content in H_2_O_2_ injury and MSC-CM treatment groups. Control group = 1. Mean ± SD. n = 10. * *P* < 0.05 vs. control group; # *P* < 0.05 vs. H_2_O_2_ group

The Bcl-2 family plays a role in the regulation of apoptosis and mitochondrial membrane potential. To address whether H_2_O_2_ injury and the protective role of MSC-CM also influence the Bcl-2 family proteins, the western blotting of Bax,Bcl-2, and BMF were assessed for the control, H_2_O_2_ and H_2_O_2_ + MSC-CM groups. Our results showed that H_2_O_2_ decreased the Bcl-2/Bax ratio significantly in comparison with the control, while MSC-CM remarkably restored this ratio. Consistent with H_2_O_2_-dependent apoptosis activation, BMF expression was markedly increased in the H_2_O_2_ group, while it was low in the control group. However, BMF expression was significantly lower in the MSC-CM group compared to that in the H_2_O_2_ group (Fig. 3, *P* < 0.05).

### Protective effect of MSC-CM on proliferation and cell cycle of L02 cells exposed to H_2_O_2_

**Fig. 4:**
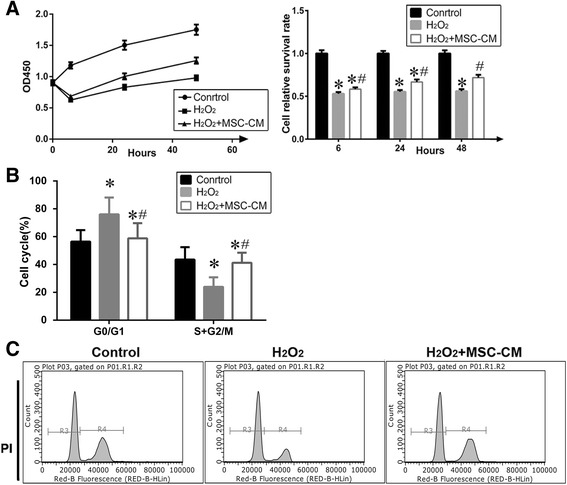
MSC-CM promoted cell viability and regulated cell cycle of the L02 cells with H_2_O_2_ injury. **a**. Effects of MSC-CM on cell viability and analysis. According to the analysis of CCK8, the optimal MSC-CM cultivated time was 24 h. (*n* = 10, Control group = 1). **b**-**c**. Cell cycle distribution in L02 cells and analysis. (Mean ± SD. *n* = 10. * *P* < 0.05 vs. control group; # *P* < 0.05 vs. H_2_O_2_ group)

H_2_O_2_ induced injury significantly reduced L02 cell viability at 24 and 48 h as compared with the H_2_O_2_ + MSC-CM group. Moreover, the cell viability of H_2_O_2_ group recovered to about 11.5% ± 2.85 and 15.8% ± 2.79 when MSC-CM was administered at 24 and 48 h, respectively (Fig. 4a, *P* < 0.05). As shown in Fig. 4b-c, H_2_O_2_ injury also resulted in a marked increase in the percentage of the cell population in the G0/G1 phase and a sharp decrease in the S and G2/M phases, indicating that H_2_O_2_ injury induces cell cycle arrest of L02 cells at the G1/S transition and the G1/S transition was pronouncedly disinhibited after treatment with MSC-CM (20% MSC-CM for 24 h, *P* < 0.05).

### Verification of miR143 and prediction of downstream target genes of miR143

**Fig. 5:**
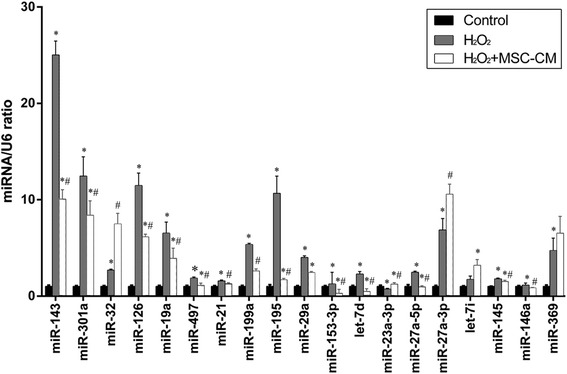
miRNA expression in L02 cells after H_2_O_2_ injury and MSC-CM treatment was validated by qRT-PCR. Control group = 1. Mean ± SD, *n* = 10, *P < 0.05 vs. control group; # *P* < 0.05 vs. H_2_O_2_ group

According to the microarray, we selected 19 disparately expressed miRNA (see Additional file 1) for RT-PCR. As shown in Fig. 5, miR143 level was significantly increased in the H_2_O_2_ group and decreased in the H_2_O_2_ + MSC-CM group. To further study the regulatory mechanism of miR143 in H_2_O_2_injury, we used the bioinformatics tools miRbase, TargetScan, and PicTar to predict target genes. ADRB1 (beta-1-adrenergic receptor) and HK2 (Hexokinase 2) were identified as candidate target genes of miR143 and had the highest predicted context score percentiles and association with glycometabolism and inflammation regulation.

### Effects of miR143 on HK2 and ADRB1 expression

**Fig. 6:**
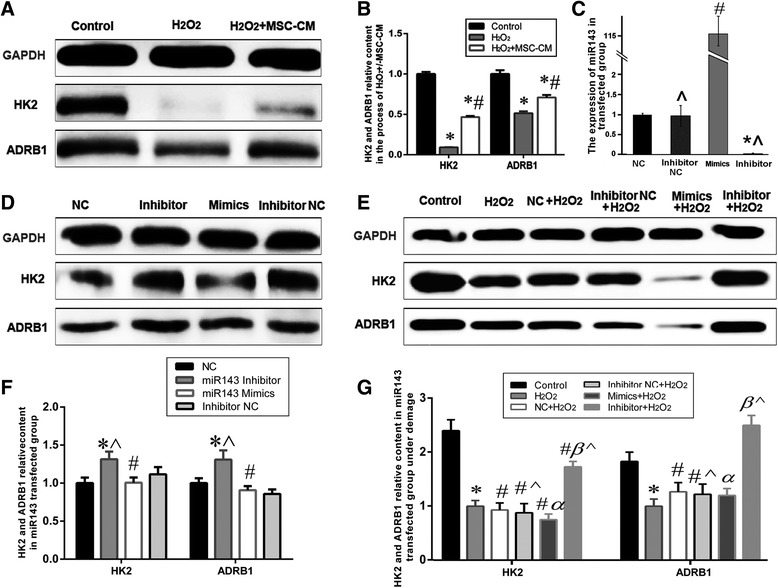
miRNA143 target proteins relative content examined by western blot analysis. **a**-**b**. HK2 and ADRB1 level decreased after H_2_O_2_ injury, and increased after MSC-CM treatment (Control group = 1, * *P* < 0.05 vs. control group; # *P* < 0.05 vs. H_2_O_2_ group). **c**. The expression of miR143 in transfected group. **d** and **f**. The expression of HK2 and ADRB1 in miRNA transfected 60 h group(NC: miRNA negative control, NC = 1, * *P* < 0.05 vs. Inhibitor NC group; # *P* < 0.05 vs. NC group; ^ denotes *P* < 0.05: Inhibitor group vs. Mimics group or Inhibitor NC group vs. NC group). **e** and **g**. The expression of HK2 and ADRB1 in the siRNA group under H_2_O_2_ injury (H_2_O_2_ group: L02 incubated with 0.8 mM H_2_O_2_ for 2 h, siRNA + H_2_O_2_ group: H_2_O_2_ treatment following siRNA transfected 60 h; H_2_O_2_ group = 1, * *P* < 0.05 vs. control group; # *P* < 0.05 vs. H_2_O_2_ group; α *P* < 0.05 vs. NC+ H_2_O_2_ group; β *P* < 0.05 vs. Inhibitor NC+ H_2_O_2_ group; ^ denotes *P* < 0.05: Inhibitor + H_2_O_2_ group vs. Mimics + H_2_O_2_ group or Inhibitor NC+ H_2_O_2_ group vs. NC+ H_2_O_2_ group). All values were expressed as mean ± SD of ten independent experiments

To further explore the role of miR143 predicted downstream target proteins HK2 and ADRB1 in MSC-CM amelioration of H_2_O_2_ injury (1 mM H_2_O_2_ for 3 h), western blot analysis was applied. The expression of HK2 and ADRB1 was significantly decreased in the H_2_O_2_ group compared to that of the control group, whereas it was significantly increased in the H_2_O_2_ + MSC-CM group (Fig. 6a–b, *P* < 0.05). Furthermore, the levels of HK2 and ADRB1 decreased in the miR143 mimics group, but increased in the inhibitor group for the L02 cells under normal incubation (Fig. 6d and f, *P* < 0.05) and the cells incubated with 0.8 mM H_2_O_2_ for 2 h after miRNA transfection for 60 h (Fig. 6e and g, *P* < 0.05). It is worth mentioning that the difference in HK2 and ADRB1 expression levels between the miR143 mimics group and the inhibitor group markedly increased under H_2_O_2_ injury.

### Effects of miR143 on L02 apoptosis after transfection for 60 h

**Fig. 7:**
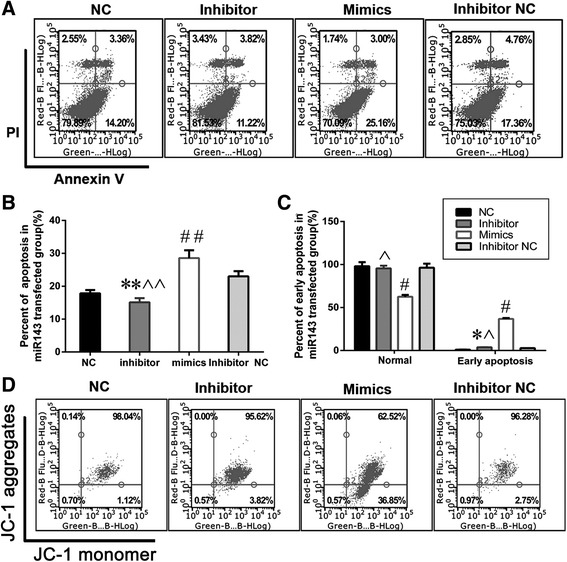
miR143 promotes L02 apoptosis after transfection for 60 h. **a**-**b**. miR143 accelerated L02 apoptosis examined by Annexin V/PI double staining and flow cytometry. **c**-**d**. miR143 aggravated L02 MMP depolarization examined by JC-1 staining and flow cytometry. The lower right quadrant: MMP depolarization. (*n* = 10, *P < 0.05 and **P < 0.01 vs. Inhibitor NC group; #*P* < 0.05 and ##*P* < 0.01 vs. NC group; ^*P* < 0.05 and ^^*P* < 0.01 vs. Mimics group)

**Fig. 8:**
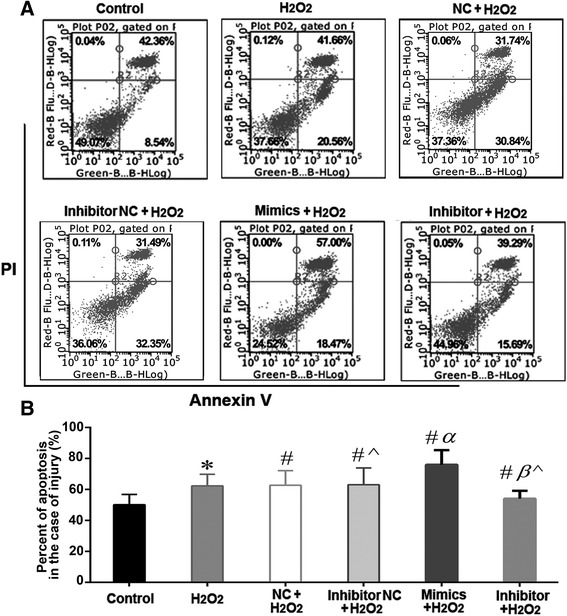
miR143 promotes L02 apoptosis in the case of H_2_O_2_ injury. **a**-**b**. miR143 overexpression accelerated L02 apoptosis, down regulated miR143 protected L02 cells from apoptosis (The lower left quadrant represents normal cells). n = 10, * *P* < 0.05 vs. control group; # *P* < 0.05 vs. H_2_O_2_ group; α *P* < 0.05 vs. NC+ H_2_O_2_ group; β *P* < 0.05 vs. Inhibitor NC+ H_2_O_2_ group; ^ denotes *P* < 0.05: Inhibitor + H_2_O_2_ group vs. Mimics + H_2_O_2_ group or Inhibitor NC+ H_2_O_2_ group vs. NC+ H_2_O_2_ group

According to the flow cytometric analysis with AnnexinV/PI staining, the percentage of apoptotic cells was higher in the miR143 mimics group compared to the inhibitor group (28.59% ± 2.36% vs. 15.13% ± 1.27%, Fig. 7a–b, *P* < 0.05). Alternatively, JC-1 staining showed the percent of cells with mitochondrial membrane potential depolarization was 3.85% ± 0.15% in the miR143 inhibitor group, while it was 36.78% ± 1.15% in the miR143 mimics group (Fig. 7c–d, *P* < 0.05). In the case of H_2_O_2_ induced injury, the percentage of apoptotic cells reached 54.98% ± 5.05% and 75.47% ± 9.43% in the miR143 inhibitor and miR143 mimics groups, respectively. The apoptosis rate of the inhibitor NC + H_2_O_2_ group was 63.09% ± 10.87% and for the NC + H_2_O_2_ group it was 62.74% ± 9.47% (Fig. 8a–b, *P* < 0.05).

**Fig. 9:**
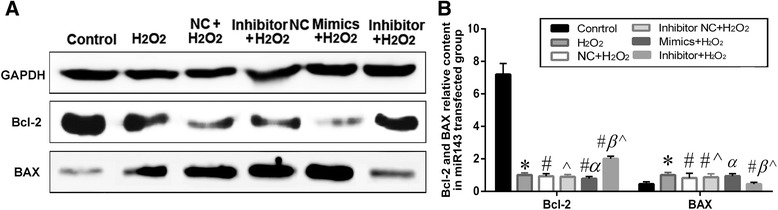
Expression of apoptosis-related proteins. **a**. western blot analysis shows the relative contents of Bcl2 and Bax. **b** The quantified histogram of western blot images. H_2_O_2_ group = 1, n = 10, * *P* < 0.05 vs. control group; # *P* < 0.05 vs. H_2_O_2_ group; α *P* < 0.05 vs. NC+ H_2_O_2_ group; β *P* < 0.05 vs. Inhibitor NC+ H_2_O_2_ group; ^ denotes *P* < 0.05: Inhibitor + H_2_O_2_ group vs. Mimics + H_2_O_2_ group or Inhibitor NC+ H_2_O_2_ group vs. NC+ H_2_O_2_ group

Apoptosis-related proteins were utilized to reflect cellular apoptosis. In order to further investigate the function of miR143, expression levels of Bcl-2 and Bax were detected in the miR143-transfected group with H_2_O_2_injury (0.8 mM H_2_O_2_ for 2 h). Bax expression was significantly increased in the miR143 mimics + H_2_O_2_ group, and was low in the control group and NC + H_2_O_2_ group. However, Bax expression was significantly lower in the inhibitor + H_2_O_2_ group compared to that in the inhibitor NC + H_2_O_2_ and control groups. On the other hand, Bcl-2 expression was significantly decreased in the miR143 mimics + H_2_O_2_ group compared to that in the NC + H_2_O_2_ and H_2_O_2_ groups, whereas it was significantly higher in the inhibitor + H_2_O_2_ group compared to that in the inhibitor NC + H_2_O_2_ and H_2_O_2_ groups. In the H_2_O_2_ injury group, Bcl-2 expression decreased, while Bax expression increased in both the up-regulation and down-regulation of miR143. However, a much greater decrease of Bcl-2 expression levels and increase of Bax levels were observed with the up-regulation of miR143, compared with miR143 down-regulation. Furthermore, the Bcl-2/Bax ratio was significantly reduced in the miR143 mimics group compared to the inhibitor group (Fig. 9a–b, *P* < 0.05). These changes in Bax and Bcl-2 were consistent with H_2_O_2_ decreased the Bcl-2/Bax ratio significantly in comparison with the control, while MSC-CM remarkably restored this ratio under the condition of H_2_O_2_ injury and MSC-CM treatment. These results indicated that miR143 was associated with apoptosis.

### Effects of miR143 on L02 cells proliferation and cell cycle

**Fig. 10:**
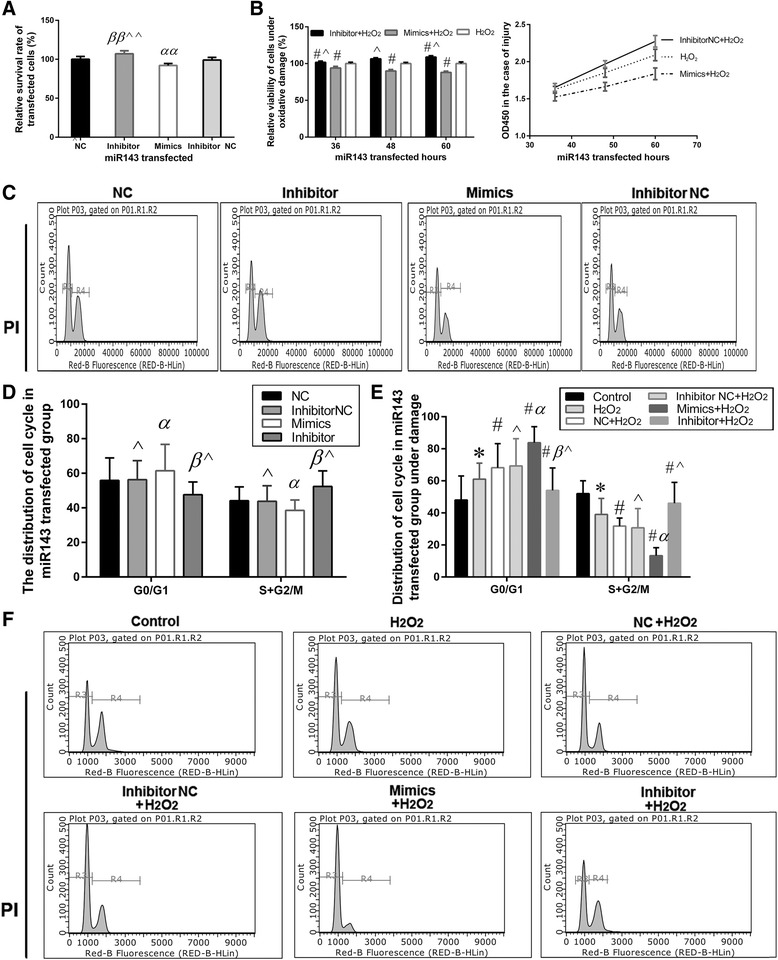
Effects of miR143 on L02 cell proliferation and cell cycle. **a** Effects of miR143 on cell proliferation and analysis (NC group = 1, αα *P* < 0.01 vs. NC group; ββ *P* < 0.01 vs. Inhibitor NC group; ^^ *P* < 0.01 vs. Mimics group). **b**. Effects of miR143 on cell proliferation and analysis under H_2_O_2_ injury (H_2_O_2_ group = 1, # *P* < 0.05 vs. H_2_O_2_ group; ^ *P* < 0.05 vs Mimics + H_2_O_2_ group). **c**-**d**. Cell cycle distribution in L02 cells and analysis. **e**-**f**. Cell cycle distribution and analysis under H_2_O_2_ injury (* *P* < 0.05 vs. control group; # *P* < 0.05 vs. H_2_O_2_ group; α *P* < 0.05 vs. NC+ H_2_O_2_ group; β*P* < 0.05 vs. Inhibitor NC+ H_2_O_2_ group; ^ denotes *P* < 0.05: Inhibitor + H_2_O_2_ group vs. Mimics + H_2_O_2_ group or Inhibitor NC+ H_2_O_2_ group vs. NC+ H_2_O_2_ group). All values were expressed as mean ± SD of ten independent experiments

According to the CCK-8 analysis, 11.21% ± 3.76% of the L02 cells were viable in the miR143 inhibitor group as compared to the mimics group at 60 h after transfection (Fig. 10a, *P* < 0.05). In addition, the viability of L02 cells increased by 14.5% ± 3.17% and 18.9% ± 3.94% at 48 and 60 h respectively in the miR143 inhibitor group as compared to the mimics group, when subjected to H_2_O_2_ injury (0.8 mM H_2_O_2_ for 2 h) following transfection (Fig. 10b, *P* < 0.05).

Meanwhile, over-expression of miR143 also resulted in a marked increase in the percentage of the cell population in the G0/G1 phase and a sharp decrease in the S + G2/M phase as compared to the down-regulation of miR143 after transfection for 60 h (Fig. 10c–d, *P* < 0.05). After H_2_O_2_ injury, a significant increase in the percentage of the cell population in G0/G1 phase was observed in the miR143 mimics group, when compared with the miR143 inhibitor. Furthermore, the percentage of cells in the S + G2/M phase in the miR143 mimics group was significantly lower than that in the inhibitor group under oxidative stress injury (Fig. 10e–f, *P* < 0.05). On the other hand, the rates of occurrence of G0/G1 and S + G2/M stages were significantly different between the miR143 inhibitor and mimics groups under H_2_O_2_ injury (Fig. 10d–e, *P* < 0.05). The results indicated that miR143 induces cell cycle arrest, which is in accordance with the alteration of cell cycle in H_2_O_2_ injury and MSC-CM treatment.

## Discussion

A growing knowledge of the relation between aberrant miRNA expression and hepatocyte injury [22, 23]. Studies suggest that MSC-CM possesses the capacity to change the expression of miRNA, leading to alteration in the cell microenvironment, and play a prominent role in cell repair [17]. In this study, the therapeutic potential of MSC-CM to alleviate H_2_O_2_ injury in hepatocytes was further examined along with the possible underlying mechanisms of this function. Our study demonstrates that MSC-CM protects hepatic L02 cells from injury by adjusting apoptosis, mitosis, proliferation, and the expression of miRNA. Current evidence indicates that miRNAs play an important role in the heart [24, 25], kidney [26], and brain [27, 28] hypoxia impairment and MSC-CM repair. RT-PCR was applied to screen differentially expressed miRNA, and miR143 was selected. The predicted target proteins of miR143 were detected by bioinformatics algorithms, and then verified by western blot. Over-expression or down-regulation of miR143 aggravated or alleviated hepatocyte apoptosis induced by H_2_O_2_, respectively. In addition to increased apoptosis, reduced cell proliferation and cell cycle progression were observed in L02 cells over-expressing miR143 after H_2_O_2_ injury. Furthermore, decreased expression of predicted miR143 target proteins, HK2 and ADRB1, and reduced Bcl-2/Bax ratio were observed upon miR143 over-expression. We postulate that miR143 inhibits Bcl-2 expression and activates Bax and caspase-9 and thereby the endogenous mitochondrial pathway, which promotes cell apoptosis. Zhang’s research indicated that Bcl-2 might be a target gene of miR143 [29]. In addition, miR143 may target HK2 [30] and ADRB1 and play an important role in proliferation and apoptosis during H_2_O_2_ induced injury.

However, it has been identified that miR143 suppresses tumor cell growth and migration, silencing the *KRAS* (V-Ki-ras2 Kirsten rat sarcoma viral oncogene homolog) gene, and adjusting glucose metabolism via the miR143/HK2 axis [30, 31]. Notably, recent studies found that miR143 is an essential regulator of cancer glycolysis via targeting HK2 in lung [32], liver [33], and breast [34] cancer, and glioblastoma multiform cells [35]. However, the function of miR143 in glycolysis in liver cells during hypoxia has not been fully understood. In this study, we found that both H_2_O_2_ injury and up-regulation of miR143 can increase the expression level of miR143 but decrease the levels of HK2 and ADRB1. These results provide new evidence that hypoxia participates in the regulation of miR143 expression, indicating that reduction of HK2 and ADRB1 expression is responsible for the change of miR143 under H_2_O_2_ injury. Prominently, we speculated that miR143 exerts its glycometabolism function in hepatocyte injury by specifically targeting HK2. In addition, several studies have indicated that binding HK2 to the mitochondrial membrane accelerates the Warburg effect, which is characterized by adjusting glucose metabolism [36, 37]. Hexokinase (HK) 2 is a pivotal enzyme in glucose metabolism and catalyzes the irreversible rate-limiting step in the glycolytic pathway [38]. Recent studies have demonstrated that over-expression of HK2 has been observed in human liver cancer and it is associated with poor overall survival in patients [33]. The results of the present study demonstrated that miR143 is often up regulated and expression of HK2 is down regulated in L02 cells under H_2_O_2_ injury. These results indicate that miR143 may directly target the 3’-UTR of HK2, thereby suppressing glucose consumption, lactate production, cellular *G6P* and *ATP* levels, and cell proliferation of liver cells under H_2_O_2_ injury. MSC-CM contains large numbers of cytokines, promoting cell growth after H_2_O_2_ is removed [14–16], and MSC-CM can provide a biosynthetic advantage for cell proliferation. It is reasonable to suggest that MSC-CM may have the potential to affect L02 cells subjected to H_2_O_2_ injury by reparation through adjusting glycolysis. In short, the mechanism of promoting hepatic L02 cell proliferation by regulating the miR143/HK2 axis in the MSC-CM protected group is consistent with previous studies.

Several β adrenoceptor antagonists have been shown to provide brain protection in in-vivo studies, Goyagi et al. have been confirmed [39], while, stimulation of β-adrenergic receptors increases cardiac myocyte apoptosis in vivo and in vitro [40–43]. Recent research shows that augmented hepatic β-AR signaling during aging may increase lipid accumulation in the liver [44, 45]. Hence, the major findings of our study are that H_2_O_2_ injury and miR143 over-expression decreased ADRB1 expression, but this was restored following MSC-CM treatment and miR143 down-regulation. We speculate that miR143 alleviates H_2_O_2_-induced apoptosis through specifically activating ADRB1 under hypoxia. Some research showed that during ischemia, *PKA* activation causes inactivation of cytochrome-*c* oxidase and contributes to myocardial damage due to ischemia-reperfusion. It is speculated that β-adrenergic stimulation during ischemia activates *PKA* via endogenous catecholamine release [46]. Hence β-adrenergic stimulation may mediate both myocardial protection and damage during ischemia. ADRB1 plays a critical protective role in hypoxia/reoxygenation-induced neonatal rat cardiomyocyte injury in vitro [47]. Swift et al. indicated that ADRB1 agonist treatment during dobutamine disuse mitigates negative changes in cancellous bone microarchitecture and inhibits increases in osteocyte apoptosis [48]. In addition, Zapater et al. proposed that Beta-adrenergic receptor-1 antagonist accelerates liver norepinephrine modulation of the pro-inflammatory response in CCl(4)-treated mice [49]. ADRB1 expression was restored after MSC-CM treatment. It is possible that the ADRB1 protection mechanism also contributes to the reparation of L02 cells. According to the results, we speculated that miR143 attenuates H_2_O_2_-induced hepatocyte injury following MSC-CM treatment through specifically activating ADRB1.

## Conclusions

In summary, this study showed that MSC-CM has dramatic potential to alleviate H_2_O_2_ induced oxidative stress injury and demonstrated for the first time that miRNA is involved in the molecular mechanism of the therapeutic effect of MSC-CM on hepatocyte injury. Interestingly, miR143, via adjusting downstream target proteins, was also involved in the molecular mechanism underlying the therapeutic effect of MSC-CM on oxidative stress injury by adjusting the expression of HK2 and ADRB1. Future study endeavors will be required to probe into the precise regulatory mechanisms of how miRNAs exert their functions and elucidate the signaling pathways targeted by miRNAs in MSC-CM protected hepatocytes during H_2_O_2_ induced apoptosis and necrosis.

## Additional file

Additional file 1: The 19 miRNAs with significant differences in expression in cirrhotic rats treated with MSC. *P* < 0.05. (DOCX 12 kb)

## Abbreviations

ADRB1: Beta-1 adrenergic receptor
BMF: Modifying factor
FBS: Fetal bovine serum
GAPDH: Glyceraldehyde 3-phosphate dehydrogenase
H_2_O_2_: Hydrogen peroxide
HK2: Hexokinase II
HRP: Horseradish peroxidase
miRNA: microRNA
MSC: Mesenchymal stem cell
MSC-CM: Mesenchymal stem cell-conditioned medium
OD: Optical density
PCR: Polymerase chain reaction
PI: Propidium iodide
RT: Reverse transcriptase
