# Supplementary material for: Mesenchymal stem cell conditioned medium alleviates oxidative stress injury induced by hydrogen peroxide via regulating miR143 and its target protein in hepatocytes
Source: BMC Immunol. 2017 Dec 19;18:51. doi: 10.1186/s12865-017-0232-x (PMC5735881; doi:10.1186/s12865-017-0232-x)
Supplement: Additional file 1: — The 19 miRNAs with significant differences in expression in cirrhotic rats treated with MSC. P < 0.05. (DOCX 12 kb) [file 12865_2017_232_MOESM1_ESM.docx]

**Additional file 1. The 19 miRNAs with significant differences in expression in cirrhotic rats treated with MSC**. P<0.05.

| **Name** | **Nor vs CTr** | **MSC vs CTr** | **Name** | **Nor vs CTr** | **MSC vs CTr** |
| --- | --- | --- | --- | --- | --- |
| **miR-369** | 80.111475 | 147.2969979 | **miR-301a** | 0.121209718 | 0.19035828 |
| **miR-23a-3p** | 36.98776384 | 29.78073715 | **miR-145** | 0.378127432 | 0.199238652 |
| **miR-153-3p** | 114.0289079 | 23.93032015 | **miR-497** | 0.043155987 | 0.201633348 |
| **miR-27a-3p** | 30.36150649 | 7.183449651 | **miR-126** | 0.075343361 | 0.203989708 |
| **miR-27a** | 0.240504722 | 0.092909484 | **miR-146a** | 0.082396386 | 0.264096021 |
| **let-7i** | 0.202612793 | 0.102153412 | **let-7d** | 0.055460608 | 0.281104311 |
| **miR-199a-3p** | 0.034090233 | 0.137057962 | **miR-21** | 0.048171714 | 0.370736084 |
| **miR-195** | 0.054897944 | 0.162361701 | **miR-29a** | 0.094114642 | 0.400061078 |
| **miR-32** | 0.026715611 | 0.205586943 | **miR-143** | 0.031378242 | 0.419264369 |
| **miR-19a** | 0.049980208 | 0.175824913 |  |  |  |
